# The Effects of Annealing Temperatures on Composition and Strain in Si*_x_*Ge_1−_*_x_* Obtained by Melting Growth of Electrodeposited Ge on Si (100)

**DOI:** 10.3390/ma7021409

**Published:** 2014-02-24

**Authors:** Mastura Shafinaz Zainal Abidin, Tahsin Morshed, Hironori Chikita, Yuki Kinoshita, Shunpei Muta, Mohammad Anisuzzaman, Jong-Hyeok Park, Ryo Matsumura, Mohamad Rusop Mahmood, Taizoh Sadoh, Abdul Manaf Hashim

**Affiliations:** 1 Faculty of Electrical Engineering, Universiti Teknologi Malaysia, Johor, Skudai 81310, Malaysia; 2 Malaysia-Japan International Institute of Technology, Universiti Teknologi Malaysia, Jalan Semarak, Kuala Lumpur 54100, Malaysia; E-Mail: mtahsin2@live.utm.my; 3 Department of Electronics, Kyushu University, 744 Motooka, Fukuoka 819-0395, Japan; E-Mails: h_chikita@nano.ed.kyushu-u.ac.jp (H.C.); y_kinoshita@nano.ed.kyushu-u.ac.jp (Y.K.); s_muta@nano.ed.kyushu-u.ac.jp (S.M.); maniuz@gmail.com (M.A.); j_park@nano.ed.kyushu-u.ac.jp (J.-H.P.); r_matsumura@nano.ed.kyushu-u.ac.jp (R.M.); sadoh@ed.kyushu-u.ac.jp (T.S.); 4 Faculty of Electrical Engineering, Universiti Teknologi MARA, Selangor, Shah Alam 40450, Malaysia; E-Mail: nanouitm@gmail.com; 5 MIMOS Berhad, Technology Park Malaysia, Kuala Lumpur 57000, Malaysia

**Keywords:** germanium, silicon, electrochemical deposition, rapid melting

## Abstract

The effects of annealing temperatures on composition and strain in Si*_x_*Ge_1−_*_x_*, obtained by rapid melting growth of electrodeposited Ge on Si (100) substrate were investigated. Here, a rapid melting process was performed at temperatures of 1000, 1050 and 1100°C for 1 s. All annealed samples show single crystalline structure in (100) orientation. A significant appearance of Si-Ge vibration mode peak at ~00 cm^−1^ confirms the existence of Si-Ge intermixing due to out-diffusion of Si into Ge region. On a rapid melting process, Ge melts and reaches the thermal equilibrium in short time. Si at Ge/Si interface begins to dissolve once in contact with the molten Ge to produce Si-Ge intermixing. The Si fraction in Si-Ge intermixing was calculated by taking into account the intensity ratio of Ge-Ge and Si-Ge vibration mode peaks and was found to increase with the annealing temperatures. It is found that the strain turns from tensile to compressive as the annealing temperature increases. The Si fraction dependent thermal expansion coefficient of Si*_x_*Ge_1−_*_x_* is a possible cause to generate such strain behavior. The understanding of compositional and strain characteristics is important in Ge/Si heterostructure as these properties seem to give significant effects in device performance.

## 1. Introduction

Remarkable progresses in the scaling down of silicon metal-oxide-semiconductor field-effect transistor (Si-MOSFET) with the introduction of several innovations such as strained Si [1], high-*k* materials [2–4], and tri-gate [4–6] structure have enhanced the performance of Si ultra-large-scale integrated circuits (ULSIs). However, further down-scaling of Si-MOSFET seems to be impossible due to several severe problems like short channel effect [7], gate leakage current [7,8], *etc.* The most promising alternative solution is to replace Si with new channel materials such germanium (Ge) that possess higher carrier mobilities so that the performance of conventional complementary metal oxide semiconductor (CMOS) transistors can be enhanced [9,10]. A co-integration of Ge on Si platform should enable the realization of the so-called “More than Moore” technology [10] where this material can not only be used for the fabrication of high speed CMOS transistor but also for the fabrication of other functional devices, such as sensors [11], photodetectors [12,13], optical devices [14], solar batteries [15], display panels [16,17], *etc.* Nowadays, there is a great deal of research on the growth of Ge on Si [18–22], which seems to accelerate the realization of such technology. As a result, a co-integration of Ge on Si platform, *i.e.*, Ge/Si heterostructure, seems to offer the present ultra-large-scale-integrated circuits (ULSIs) with superb multi-functionalities [10].

The growth of Ge on Si can be performed by several techniques. In general, high vacuum methods, such as chemical vapor deposition (CVD) [20,21,23] and molecular beam epitaxy (MBE) [24–26], are used for the deposition. Whereas, low vacuum techniques, such as sputtering and thermal or electron beam evaporation, are not able to keep the surface from being contaminated during the deposition, which normally affect the subsequent crystallization process [18,19,27]. A low cost and simple technique for the deposition of Ge films by an electrochemical technique seems to offer more advantages in term of the controllability of growth parameters and quality [18,28]. In general, electrolytes play an important role in determining the quality of the electrodeposited films. It has been intensively reported that the electrodeposition of Ge on semiconductor substrate is mainly achieved in non-aqueous solvent, such as ionic liquid [29–36]. For example, Freyland *et al.* [36] reported the electrodeposition of Ge on hydrogenated (H)–Si (111) using ionic molten salt, [BMIm]^+^PF_6_^−^ saturated with germanium (IV) tetrachloride, GeCl_4_ or germanium (IV) bromide, GeBr_4,_ at room temperature. Al-Salman *et al.* [33] demonstrated the direct electrodeposition of Si*_x_*Ge_1−_*_x_* structure using ionic liquid. In general, the as-deposited Ge layer shows an amorphous structure. The crystallization of deposited Ge can be easily achieved by applying a so-called rapid melting process [18,37]. By applying such high annealing temperatures, the intermixing Si-Ge was observed at Ge/Si interface [38–40]. To date, a great deal of intensive theoretical and experimental studies related to Ge/Si interface have been reported [27,39,41,42]. However, those reported Ge/Si structures were mostly grown by CVD and MBE system.

Recently, we demonstrated the electrodeposition of Ge films on Si (100) by using an electrolyte formed by a mixture of GeCl_4_ and propylene glycol, C_3_H_8_O_2_ and their subsequent crystallization process was successfully achieved by rapid melting process at 980°C for 1 s [40]. It has been observed that the Si-Ge intermixing occurred at Ge/Si, as indicated by Raman spectra and depth profile analysis. In this paper, we further report the effects of annealing temperatures on the Si-Ge intermixing of electrodeposited Ge, particularly, the studies on the composition and strain. Different composition of Ge and Si, also its strain, were established by applying different rapid melting temperature on electrodeposited Ge on Si. The understanding of Ge and Si compositional characteristics and its strain is important in Ge/Si heterostructure since these properties seem to give significant effects in device applications [43–45].

## 2. Experimental Section

A deposition of Ge layer on Si (100) substrate was performed by an electrochemical process. Prior to deposition, Si substrates were cleaned by standard RCA process and diluted hydrofluoric (HF) acid to remove native oxide layer. The deposition was carried out in a mixture of 5% GeCl_4_ in C_3_H_8_O_2_ where Si substrate was set as a cathode and Pt wire as an anode [40,46]. The process was done at room temperature with the applied current density of 20 mA/cm^2^ for 30 min. Then, the samples were immersed in the deionized (DI) water after the deposition.

**Figure 1.**
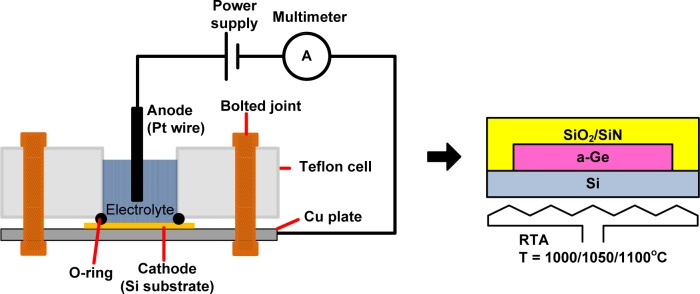
Schematic representations of electrochemical deposition setup and rapid melting process.

Subsequently, the as-deposited Ge films (thickness = 80 nm) were patterned into circular shape with diameter, φ of 20 μm using photolithography and wet etching. A capping layer of SiO_2_/SiN*_x_* with total thickness of 2.5 μm was deposited by magnetron sputtering. The patterning of Ge into circular shape and deposition of capping layer were applied to prevent the Ge agglomeration during the annealing process [27,47]. The samples were annealed at various temperature, from 1000 to 1100°C, for 1 s, to crystallize the Ge structure. The annealing process was carried out using a rapid thermal furnace (MILA 3000, ULVAC-RIKO Inc., Kanagawa, Japan). The temperature was ramped up in two stages. At the first stage, the temperature was ramped up from room temperature to 800°C in 1 min. Then, the heating was kept at 800°C for 1 min. It was followed by the second stage where the temperature was ramped up from 800°C to the set temperatures, *i.e.*, 1000, 1050, and 1100°C in 5 s. By applying such a two stage of temperature ramping procedure, the temperature overshoot can be avoided and also the difference between the setting temperature and actual temperature can be minimized. The furnace was programmed to be turned off immediately when reaching the set temperature. The system was cold down naturally in N_2_ flow. The capping layer was removed prior to characterization. The schematic representation of experimental works is shown in Figure 1.

The morphology, crystal orientation, structural properties, and depth profiles of the samples were characterized using Nomarski microscopy (BX51M, Olympus Corp., Tokyo, Japan), electron backscattering diffraction (EBSD, EBSP JSM-5510LS scanning electron microscopy, JEOL Ltd., Tokyo, Japan), Raman spectroscopy (Horiba Jobin Yvon, Ar^+^ laser, 514 nm wavelength, 20 mW power, Horiba, Japan), and Auger electron spectroscopy (AES, JAMP-9500F, accelerating voltage: 10 kV, JEOL Ltd.).

## 3. Results and Discussion

**Figure 2.**
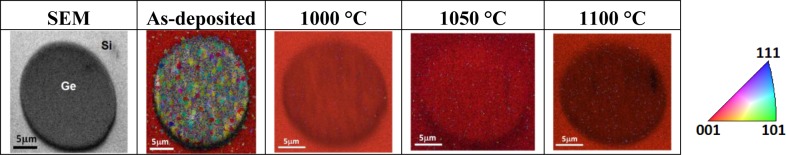
SEM and EBSD images of annealed Ge/Si at different temperatures.

Figure 2 shows the EBSD images of annealed Ge/Si. Based on colour coded representations, the crystallographic orientation of grown samples can be easily determined. It can be seen clearly that, random distribution colors are obtained for as-deposited samples that indicates amorphous structure. This structure was further characterized by Raman spectroscopy, where broad and low intense peak for Ge-Ge vibration mode was obtained. On the other hand, uni-color codes for all annealed samples were obtained. This shows that crystallization was achieved and the orientation of grown Ge were confirmed to be in (100) which similar to that of Si (100) orientation as indicated by a solid red color. EBSD mapping or image can also be used to roughly understand the surface morphology of the material based on the brightness of generated color. In general, the color of all images for samples grown at 1000, 1050, and 1100°C were confirmed to be red. However, it can be seen that the images slightly turn dark red in color with the increase of temperature. This simply suggests that the roughness of the grown structure increases with the temperature.

**Figure 3.**
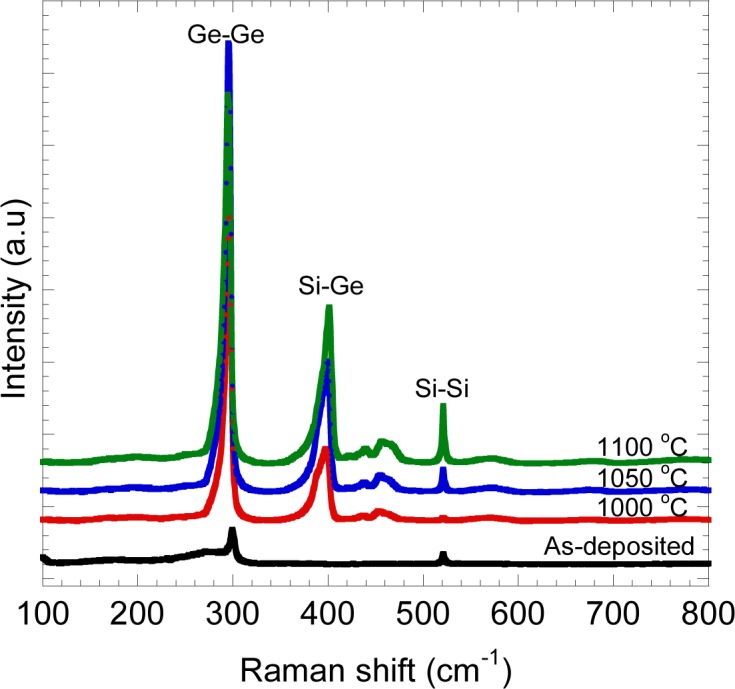
Typical Raman spectra of annealed Ge/Si at different temperatures.

**Figure 4.**
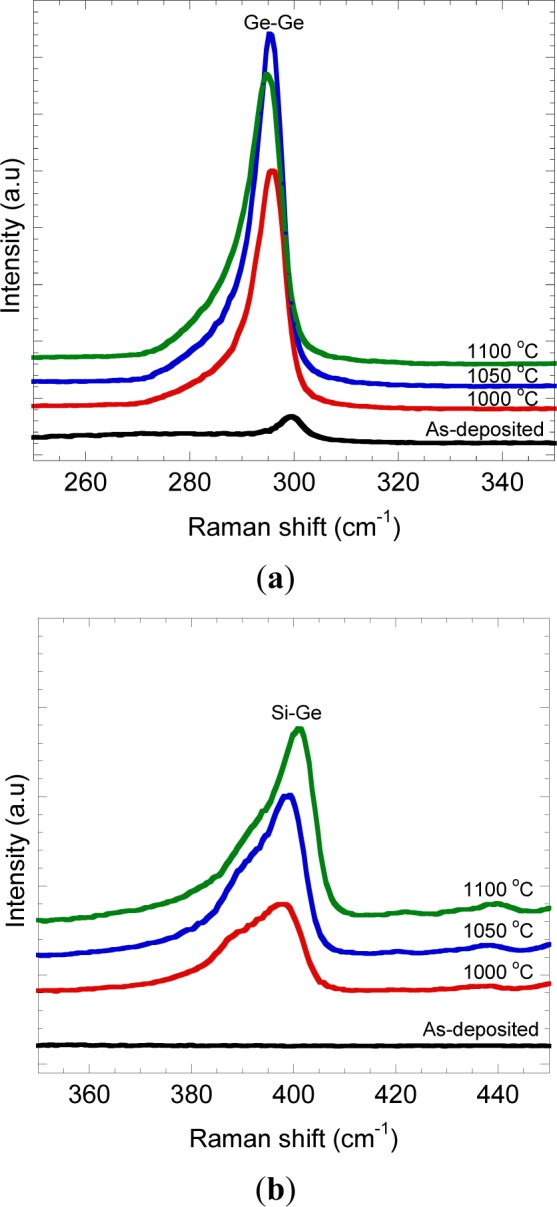
Enlarged Raman spectra in the range of (**a**) Ge-Ge mode and (**b**) Si-Ge mode.

The typical Raman spectrum is shown in Figure 3. The highly intense peak at ~00 cm^−1^ indicating Ge-Ge vibration mode was significantly observed for all annealed samples. This indicates that the crystallization of deposited Ge on Si was obtained by rapid melting process. In addition to that, a sub peak at ~00 cm^−1^ corresponds to Si-Ge vibration mode was also observed, thus, confirming the existence of Si-Ge intermixing at the Ge/Si interface. On rapid melting process, Ge melts and reaches the thermal equilibrium in a short time. Si at the Ge/Si interface begins to dissolve once in contact with the molten Ge to produce Si-Ge intermixing and result in Si*_x_*Ge_1−_*_x_* (0 ≤ *x* ≤ 1) formation [41,48]. By further observing the peak of Ge-Ge and Si-Ge vibration modes, it was found that the frequency, ω was slightly shifted as indicated in Figure 4.

In Si*_x_*Ge_1−_*_x_* system, ω is mainly affected by several parameters, namely, (i) the content of Ge and Si [49] and (ii) strain [49]. In unstrained Si*_x_*Ge_1−_*_x_* system, the Raman peak position may solely be determined by the composition, but both parameters need be taken into account while considering for strained Si*_x_*Ge_1−_*_x_*. Raman spectroscopy has been reported to be an effective quantitative evaluation of composition and strain [49,50]. Thoroughly, the ratio of composition can be determined by referring to the relative intensities of the first order of Ge-Ge, Si-Ge and Si-Si Raman modes [51]. The peaks shift to higher ω in Si-Ge mode may indicate the change of epilayer to be more compressive [49,50].

**Figure 5.**
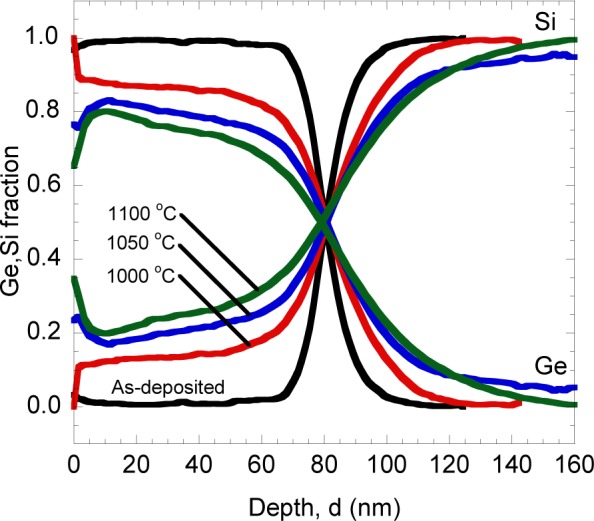
Depth profiles of samples annealed at different temperatures.

The AES measurement was performed to investigate the relationship between the Si-Ge intermixing and the thickness of Ge/Si. Figure 5 shows the comparison of depth profiles of as-deposited and annealed samples. In all annealed samples, it is found that the intermixing not only occurred at the Ge/Si interface, but Si seems to diffuse up to nearly the top surface of the Ge/Si structure. The composition of Si in Ge region increases with the annealing temperature as expected. Thus, it can be concluded that temperature of rapid melting process is one of the significant parameter in determining the intermixing ratio of Si-Ge.

Based on Raman spectra, the composition and strain can be determined since strained Si*_x_*Ge_1−*x*_ is considered in this study. By taking into account the intensity ratio of Ge-Ge and Si-Ge vibration mode peaks, the Si fraction (*x*: 0 ≤ *x* ≤ 1) in the surface regions of grown layers was calculated using Equation (1):

$$\frac{I^{\mathrm{Ge}-\mathrm{Ge}}}{I^{\mathrm{Si}-\mathrm{Ge}}}=\frac{k(1-x)}{2x}$$ (1)

**Figure 6.**
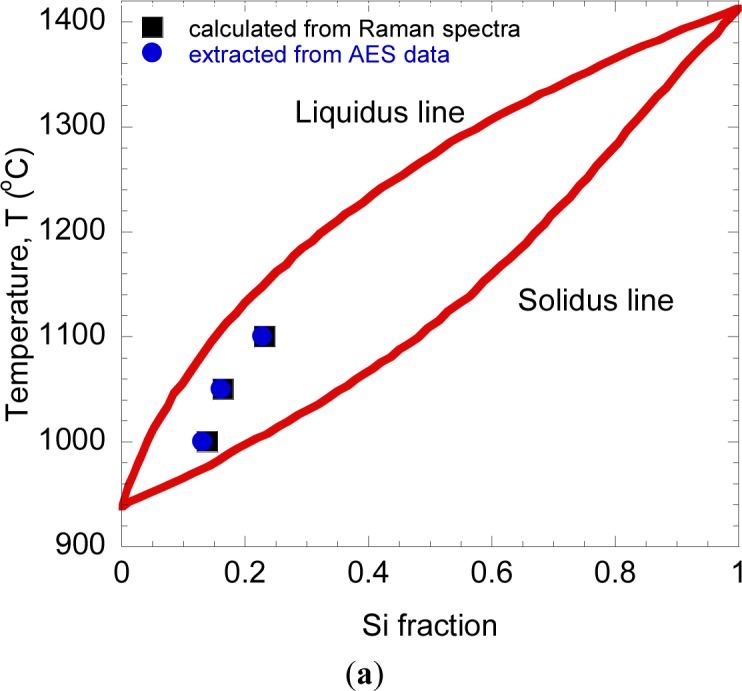

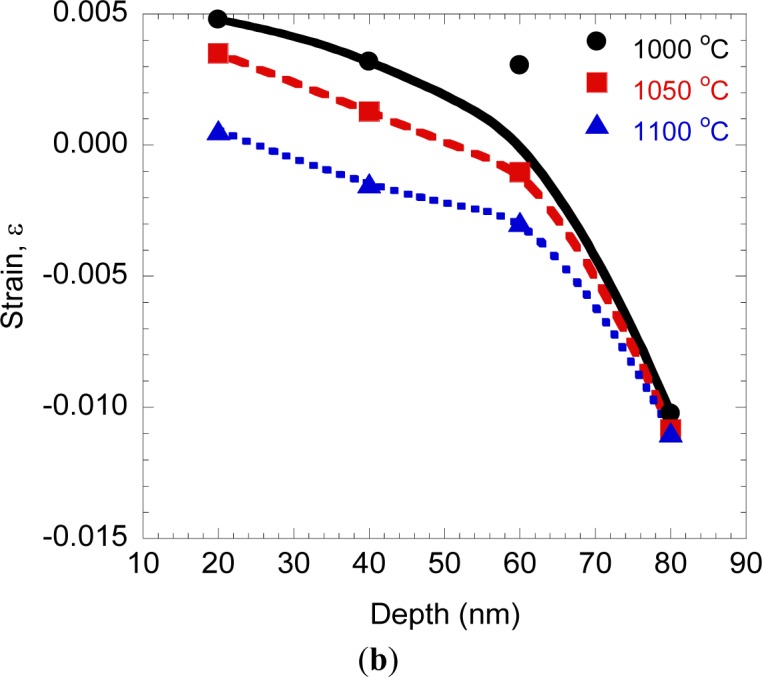
(**a**) Si fraction as a function of annealing temperatures and (**b**) strain at respective depth for different annealing temperatures.

Here, *I*^Ge-Ge^ and *I*^Si-Ge^ are the peak intensities of Raman signals originating from Ge-Ge and Si-Ge vibration modes, respectively, and k is a constant. Equation (1) was cited from Mooney *et al.* [51]. They have investigated the ratio of Raman peak intensities due to Ge-Ge and Si-Ge bonding as a function of the Si fraction. Based on the experimental data, they have derived Equation (1). Here, the constant k depends on the wavelength of exciting laser. The constant k value in our measurement system was determined as 1.6 by measuring the Raman spectra of single crystalline Si*_x_*Ge_1−_*_x_*, where the Si*_x_*Ge_1−_*_x_* (*x* = 0.11, 0.21, 0.43, 0.51) samples were epitaxially grown on Si substrates [27]. The penetration depth of Ar^+^ laser with the wavelength 514 nm is about 20 nm [52]. Therefore, these calculated Si fractions may represent the composition of Si diffused into Ge region at 20 nm depth from the surface. The peak intensity of Ge-Ge and Si-Ge peaks depend on the Si fractions in the Ge layer after rapid melting process. It is expected that the peak intensity of Ge-Ge peak should become lower, while the peak of Si-Ge peak should become higher with the increase of temperature. This is due the increase of Si fraction in Ge layer with the increase of temperature, resulting to the decrease of Ge-Ge bonding and the increase of Si-Ge bonding. Such a tendency was observed, as shown in Figure 3. As shown by Figure 6a, the calculated Si fractions were plotted in Ge-Si equilibrium phase diagram [53].

Both fractions, calculated from Raman spectra and extracted from AES, have shown a good agreement to each other and located in between of the liquidus and solidus lines. Here, it can be seen that the Si fraction increases with the increase of temperature. In our previous report [40] where an annealing was performed at 980°C (Ge thickness =160 nm), the Si fraction seems to be much lower compared to the present samples. We speculate that Ge thickness may have significant role in suppressing the diffusion of Si atoms into Ge region, thus, affecting the overall diffusion rate in Si*_x_*Ge_1−_*_x_*. The results suggest that composition ratio of Ge and Si is strongly controlled by annealing temperatures and the values may vary with the thickness of the Ge layer.

Ge/Si interface was assumed to be not fully strain-relaxed in this study. As indicated by the shifting in Ge-Ge vibration mode peaks, it is expected that strain exists with respect to annealing temperature. The strain, ε was determined by the following equation [49]:

$$\omega^{\mathrm{Ge}-\mathrm{Ge}}(x,\varepsilon )=280.3+19.4(1-x)-450\varepsilon$$ (2)

where *x* is the Si composition and ω^Ge-Ge^ is the frequency of Ge-Ge vibrational mode. The Si composition that extracted from AES data and frequency of Ge-Ge vibrational mode from Raman spectra for respective depths were utilized to determine the strain values by Equation (2). Equation (2) was derived by Pezzoli *et al.* [49]. They have analyzed the positions of Raman peaks due to Ge-Ge bonding as a function of Si fraction x and strain ε in Si*_x_*Ge_1−_*_x_* layer epitaxially grown on Si substrates. From the least square fit of the experimental data, they have derived Equation (2). Figure 6b shows the strain values as a function of depth at respective annealing temperatures. The tensile strain turns from high to low with the increase of temperatures. In addition, it drastically becomes more compressive as the depth is approaching the interface of Ge and Si. The thermal expansion coefficient of Si*_x_*Ge_1−_*_x_* is larger than that of Si [54], where the difference decreases with the increasing Si concentration. Such a fraction-dependent thermal expansion coefficient is a possible cause to generate the change in the grown Si*_x_*Ge_1−_*_x_* layers.

As reported in reference [54], the amount of strain depends on the highest temperature used in the growth/annealing. In this present study, it can be simply concluded that higher annealing temperature produce lower tensile or more compressive strain in Ge/Si structure.

From practical applications view, the presence of strain in channel will improve the transistor performance by enhancing the electron/hole mobility through reduced effective transport mass and reduced interband scattering rate [55]. It has been reported that presence of Si*_x_*Ge_1−_*_x_*, with significant levels of strain, will enhance the mobility in n-channel MOSFET (NMOS) and p-channel MOSFET (PMOS) [4,56]. Particularly, the tensile-strained Si*_x_*Ge_1−_*_x_* that integrated in NMOS transistor will induce the enhancement of electron mobility while compressive strain will induce the hole-mobility enhancement in PMOS. Further increment of Ge ratio in Si*_x_*Ge_1−_*_x_* will then affect the strain as well as the channel mobility [4,56]. For that reason, it seems that annealing temperature plays role in tuning the strain level that is significantly beneficial towards mobility enhancement of transistor applications.

## 4. Conclusions

It was shown that annealing temperature plays significant role in the crystallization of electrodeposited Ge/Si and its intermixing properties. The composition of Si and Ge fraction significantly changed due to intermixing behavior upon rapid melting process. In addition to that, the strain properties also being affected due to the changes of Si and Ge composition and significantly contribute in the improvement of carrier mobility. As a result, it is expected that this technique will facilitate high-performance CMOS transistors, as well as various functional devices.
